# Risk factors and a predictive nomogram for regional lymph node metastasis in deficient mismatch repair colorectal cancer

**DOI:** 10.3389/fonc.2025.1601993

**Published:** 2025-06-19

**Authors:** Jiawei He, Tao Wu, Maofa Gao, Yunfeng Jiao, Qiaoling Yu, Yaling Zhao, Ni Hou, Jie Li

**Affiliations:** ^1^ Department of General Surgery, the Second Affiliated Hospital of Xi’an Jiaotong University, Xi’an, China; ^2^ Department of Pathology, the Second Affiliated Hospital of Xi’an Jiaotong University, Xi’an, China; ^3^ Department of Epidemiology and Biostatistics, School of Public Health, Xi’an Jiaotong University Health Science Center, Xi’an, China; ^4^ Department of Cell Biology and Genetics, School of Basic Medical Sciences, Xi’an Jiaotong University Health Science Center, Xi’an, China

**Keywords:** colorectal cancer, mismatch repair deficiency, lymph node metastasis, clinicopathological nomogram, risk factor

## Abstract

**Objective:**

The present study aims to investigate the risk factors associated with regional lymph node metastasis (LNM) in patients with deficient mismatch repair (dMMR) colorectal cancer (CRC) and develop a nomogram for predicting it preoperatively.

**Patients and Methods:**

Clinicopathological data of patients who underwent surgical treatment at the Second Affiliated Hospital of Xi’an Jiaotong University between January 2021 and December 2024 were collected, and univariate and multivariate logistic regression analyses were performed to identify the independent risk factors for regional LNM. A clinicopathologic nomogram for preoperatively predicting LNM was established and further validated and evaluated.

**Results:**

A total of 131 patients with stage I to III dMMR/microsatellite instability (MSI) CRC were included in the study. The results showed that age, tumor location, degree of differentiation, depth of invasion, and negative immunohistochemistry staining results for MMR proteins, except for the double-negative of MLH1 and PMS2 or MSH2 and MSH6, were independent risk factors for regional LNM in dMMR/MSI CRC. They were incorporated into the individualized prediction nomogram, which showed sufficient discriminability and good calibration. Decision curve analysis indicated that the nomogram could be used for early clinical prediction of regional LNM.

**Conclusion:**

The clinicopathological nomogram, incorporating five independent risk factors, can be widely used to facilitate the preoperative prediction of regional LNM in patients with dMMR/MSI colorectal cancer, thereby developing individual treatment and improving patients’ prognoses. While the model was internally validated, further external validation is also warranted.

## Introduction

Colorectal cancer (CRC) accounts for 9.6% of cancer incidence and 9.3% of cancer-related deaths worldwide and is steadily increasing in countries undergoing major transition (1, 2). In China in 2022, new CRC cases accounted for 12.13% of the total new cancer cases (3), and the age-standard 5-year relative survival for CRC is 55.7% (4), ranking second in global cancer-related mortality (1). CRC is highly heterogeneous in its clinical and biological features, leading to striking differences in disease progression and treatment response (5). Staging by tumor node metastasis (TNM), sidedness, and molecular markers, including mismatch repair (MMR) status and RAS and BRAF mutation status, are currently regularly used in clinical practice to select patients for specific therapies (6). The microsatellite instability (MSI) phenotype is induced by deficient MMR (dMMR) function resulting from germline mutation in at least one of the MMR genes (*MLH1*, *MSH2*, *PMS2*, and *MSH6*) or epigenetic inactivation of *MLH1*. Moreover, 5%–15% of patients with CRC suffer from dMMR/MSI colorectal cancer (7), which exhibits indolent clinical behavior, including more proximal and poorly differentiated tumors, resistance to treatment with 5-FU, and better relapse-free survival (RFS) but poor overall survival after relapse (8–10). Immunotherapy significantly prolonged PFS in a first-line setting in patients with dMMR/MSI tumors (11).

A regional lymph node is the most common site of metastasis in CRC, with approximately 40% of patients with colorectal cancer having lymph node metastasis (LNM) at first visit (12). The presence of regional LNM often suggests that the tumor is more aggressive and has a high incidence of relapse and a worse prognosis (13, 14). Accurate preoperative assessment of the presence of LNM will help guide the development of an individualized treatment regimen, reduce the risk of perioperative complications, and avoid overtreatment (15). A number of studies have previously investigated the factors associated with LNM in CRC, with the aim of predicting LNM (15–17). However, the appropriate extent of lymph node dissection in CRC remains controversial (18–20). There is an urgent need to improve the accuracy of preoperative prediction of LNM in patients with CRC.

Due to the poor survival of patients with dMMR/MSI CRC after relapse and the close correlation between regional LNM and relapse, it is necessary to predict whether these patients have LNM before surgery. In this study, we conducted a retrospective analysis of the clinicopathological data of patients with dMMR/MSI colorectal cancer who underwent curative surgical treatment at our hospital from January 2021 to December 2024. The aim of our study is to identify the risk factors for LNM and predict it in patients with dMMR/MSI colorectal cancer, which could contribute to improving treatment efficacy and patient survival.

## Patients and methods

### Study population

We performed a retrospective analysis of the clinical and pathological data of patients with dMMR/MSI colorectal cancer who underwent curative resection in the Department of General Surgery of the Second Affiliated Hospital of Xi’an Jiaotong University between January 2021 and December 2024. The inclusion criteria were as follows: (1) patients whose age was ≥18 years old; (2) patients with dMMR colorectal cancer confirmed by pathology; and (3) patients who had no serious dysfunction of the heart, lungs, liver, kidneys, and other organs. The exclusion criteria were as follows: (1) patients who were diagnosed with proficient MMR (pMMR) colorectal cancer; (2) patients with primary tumors in other organs; (3) patients with distant metastasis; (4) patients who received other tumor-related treatments before surgery, such as radiotherapy and chemotherapy; and (5) patients who received palliative treatment. All patients underwent comprehensive preoperative examination, including complete physical examination, colonoscopy, pathological biopsy, chest x-ray or computed tomography (CT), abdominal and pelvic enhanced CT, and/or magnetic resonance imaging (MRI). This study was approved by the Academic Ethics Committee of Xi’an Jiaotong University Health Science Center (Permission No. 2020-002). Written informed consent was obtained from all patients.

### Data collection

Patient characteristics and histopathological variables were retrieved from the medical records. The patient characteristics consisted of age, gender, and tumor location. The tumor location was classified into three groups: right colon, left colon, or rectum. Right colon cancer was defined as a tumor located between the cecum and the transverse colon. Left colon cancer was defined as a tumor located between the splenic flexure and the sigmoid colon. Information retrieved from the postoperative histopathological reports included tumor size (presented as the longest diameter), tumor morphology (protruded type, ulcer type and infiltrative type), histological type (adenocarcinoma and mucinous adenocarcinoma), histological differentiation degree (low-grade or high-grade differentiation; low-grade differentiation included well and moderately differentiated and high-grade differentiation included poorly differentiated and undifferentiated), depth of invasion, vascular invasion, neural invasion, the presence of LNM, and MMR status, which was determined by immunohistochemistry (IHC) staining results (pMMR with positive staining of all four MMR proteins; dMMR with negative staining of at least anyone of them). Tumor stage was determined according to the TNM staging criteria of the Chinese Society of Clinical Oncology (CSCO) TNM staging system (21).

### Statistical analysis

The Pearson *χ*2 test or Fisher’s exact test was used for comparison among categorical variables that were presented as the number and percent of patients, and Student’s *t* test or the Mann–Whitney U test were used for comparison among continuous variables that were presented as the mean ± standard deviation (SD). A two-tailed *p*-value <0.05 was considered to be statistically significant. Univariate and multivariate logistic regression analyses were used to examine independent risk factors for LNM. The results of the logistic regression analyses were expressed as odds ratios (ORs) and 95% confidence intervals (CIs). The multivariate analysis included age, gender, tumor location, tumor size, tumor morphology, histological type, degree of differentiation, depth of invasion, and negative IHC staining of MMR proteins as variables, and used backward stepwise selection to screen independent risk factor variables for regional LNM in dMMR/MSI colorectal cancer. All statistical analysis were performed using IBM SPSS software (version 18.0; IBM Corp, Armonk, NY, USA) and R software (version 4.4.2; http://www.Rproject.org).

### Nomogram construction and validation

The variables with a two-tailed *p*-value lower than 0.05 in the multivariate regression analysis were used to develop a nomogram for predicting LNM in dMMR/MSI colorectal cancer. Each predictor value is represented on a separate axis, and the corresponding number of points is noted on the “point” axis. The sum of all predictor points (total point axis) is mapped to the predicted LNM probability at the bottom of the nomogram.

We validated the predictive efficiency of the prediction nomogram by testing the discrimination, calibration, and clinical utility. The apparent performance and discrimination of the model were assessed with the concordance statistic (C-statistic), which was calculated as the area under the receiver operating characteristic curve (AUROC). We also conducted the internal validation using 1,000 bootstrapped samples from the dataset to correct for optimism and quantify overfitting, which were the same size as the original dataset with replacements (22). In each bootstrap sample, we derived a prediction model, as in the original dataset. We calculated optimism as the difference in C-statistic between the original sample and the respective bootstrap sample. This was repeated for all bootstrap samples to estimate the average optimism, which was an estimate of internal validity and reflected validation for the underlying population of the data source. We constructed a calibration curve and evaluated the intercept [calibration-in-the-large (CITL)], Brier score, and calibration slope, and used the Hosmer–Lemeshow test to assess goodness of fit. Decision curve analyses (DCAs) were further conducted to assess the nomogram’s clinical value by quantifying the net benefits at different threshold probabilities (23).

## Results

### Patient disposition

The medical records of 145 patients with dMMR/MSI colorectal cancer were extracted. Among them, 14 cases were excluded due to the presence of other primary tumors or distant metastases, lack of histologically confirmed results, or incomplete medical records. A total of 131 patients with stage I to III dMMR/MSI colorectal cancer who underwent curative surgery were included in the study. The mean number of dissected lymph nodes among them was 15.97 (14.69–17.24). There were 96 patients without LNM, while 35 (26.72%) patients had LNM (**Table 1**), which was lower than the 40% LNM rate in patients with CRC (12). This is consistent with the previous reports (8, 23).

**Table 1:** Characteristics of the patients with dMMR colorectal cancer.

Patient characteristics	Cases	LNM (-)	LNM (+)	*p*-value
	*n*	(*n* = 96)	(*n* = 35)	
Age, mean ± SD	131	62.0 ± 12.9	66.7 ± 13.4	0.076
Gender, *n* (%)				
Male	61	44 (72.1)	17 (27.9)	0.781
Female	70	52 (74.3)	18 (25.7)	
Tumor location, *n* (%)				
Right colon	98	77 (78.6)	21 (21.4)	0.018
Left colon and rectum	33	19 (59.4)	14 (42.4)	

### Patient characteristics

The mean age of the 131 patients with dMMR/MSI colorectal cancer was 63 years, with mean ages of 62.0 and 66.7 years in the LNM negative [LNM (−)] and the LNM positive [LNM (+)] groups, respectively (**Table 1**). Among the 131 patients, 61 were male and 70 were female. There was no difference in the proportion of men/women in both groups. Furthermore, 74.8% (98/131) of the dMMR tumors were located in the right colon, and only 25.2% of them were located in the left colon and rectum, which was identical to previous reports (8–10). Compared with the tumors in the right colon, the tumors in the left colon and rectum had a higher proportion of LNM; that is, compared with tumors in the left colon and rectum, dMMR tumors in the right colon were less likely to develop LNM (**Table 1**).

### Pathological outcomes

There were no differences between the LNM (−) and LNM (+) groups in terms of tumor size, tumor morphology, and histological type (**Table 2**). The degree of tumor differentiation was mainly moderate, accounting for 60.3% (79/131). The proportion of LNM in high-grade differentiated dMMR tumors was higher than that in low-grade dMMR tumors (**Table 2**). Furthermore, 87.8% (115/131) of the dMMR tumors had penetrated the muscularis propria layer or the serous membrane layer of the intestinal wall (pT3–T4), with 29.6% in the LNM (+) group, which was almost higher than that in the LNM (+) group of pT1–T2 (p = 0.068) (**Table 2**). The proportion of LNM in dMMR tumors with positive vascular invasion and positive neural invasion was significantly higher than that in dMMR tumors with negative vascular invasion and negative neural invasion (**Table 2**).

**Table 2:** Pathological outcomes of the patients with dMMR colorectal cancer.

Histopathological variables	Cases	LNM (-)	LNM (+)	*p*-value
	*n*	(*n* = 96)	(*n* = 35)	
Tumor size (cm), mean ± SD	131	6.1 ± 2.4	6.3 ± 2.0	0.560
Tumor morphology, *n* (%)				
Protruded type	55	40 (72.7)	15 (27.3)	0.903
Ulcer type and infiltrative type	76	56 (73.7)	20 (26.3)	
Histological type of tumor, *n* (%)				
Adenocarcinoma	112	82 (73.2)	30 (26.8)	0.966
Mucinous adenocarcinoma	19	14 (73.7)	5 (26.3)	
Degree of tumor differentiation, *n* (%)				
Low-grade	81	68 (84.0)	13 (16.0)	<0.001
High-grade	50	28 (56.0)	22 (44.0)	
Depth of tumor invasion, *n* (%)				
T1–T2	16	15 (93.8)	1 (6.2)	0.068
T3–T4	115	81 (70.4)	34 (29.6)	
Vascular invasion, *n* (%)				
Positive	34	20 (58.8)	14 (41.2)	0.027
Negative	97	76 (78.4)	21 (21.6)	
Neural invasion, *n* (%)				
Positive	41	23 (56.1)	18 (43.9)	0.003
Negative	90	73 (81.1)	17 (18.9)	
Negative IHC staining of four MMR proteins, *n* (%)				
MLH1 alone	0	0	0	/
Others except for MLH1 negative cases	131	96 (73.3)	35 (26.7)	
PMS2 alone	19	11 (57.9)	8 (42.1)	0.101
Others except for PMS2 negative cases	112	85 (75.9)	27 (24.1)	
MSH2 alone	0	0	0	/
Others except for MSH2 negative cases	131	96 (73.3)	35 (26.7)	
MSH6 alone	5	3 (60.0)	2 (40.0)	0.609
Others except for MSH6 negative cases	126	93 (73.8)	33 (26.2)	
MLH1 and PMS2	80	62 (77.5)	18 (22.5)	0.172
Others except for MLH1 and PMS2 double-negative cases	51	34 (66.7)	17 (33.3)	
MSH2 and MSH6	22	17 (77.3)	5 (22.7)	0.643
Others except for MSH2 and MSH6 double-negative cases	109	79 (72.5)	30 (27.5)	
MLH1 and PMS2 or MSH2 and MSH6	102	79 (77.5)	23 (22.5)	0.043
Others except for MLH1 and PMS2 or MSH2 and MSH6 double-negative cases	29	17 (58.6)	12 (41.4)	

The results of MMR protein expression examined by IHC staining showed that among the 131 dMMR/MSI colorectal cancer cases, the double-negative staining of MLH1 and PMS2 was the most common, accounting for 61.07% (80/131), followed by MSH2 and MSH6 double-negative staining, accounting for 16.79% (22/131). There were 19 cases of PMS2 single-negative and 5 cases of MSH6 single-negative staining. There were no differences in these staining patterns between the LNM (−) and LNM (+) groups. However, the proportion of LNM in the double-negative groups for MLH1 and PMS2 or MSH2 and MSH6 was significantly lower than in the other cases (**Table 2**), which was in accordance with the previous studies (8, 24–27).

### Univariate and multivariate logistic regression analyses for preoperative prediction of LNM

As vascular invasion and neural invasion could not be detected preoperatively, they were excluded from the following univariate and multivariate logistic regression analyses. The univariate analysis results showed that tumor location, degree of differentiation, and negative IHC staining results of MMR proteins significantly increased the risk of LNM. Subsequently, multivariate backward stepwise logistic regression analysis was performed, initially incorporating nine variables, and finally yielding five significant factors. They were age (OR, 1.04; 95% CI, 1.01–1.08), tumor location in left colon and rectum (OR, 7.31; 95% CI, 2.21-24.15), high-grade tumor differentiation (OR, 9.53; 95% CI, 3.16–28.4), T3–T4 tumor invasion (OR, 22.20; 95% CI, 1.87–262.8), and negative IHC staining results of MMR proteins, except for double-negative staining of MLH1 and PMS2 or MSH2 and MSH6 (OR, 5.09; 95% CI, 1.56–16.67) (**Table 3**), indicating these five variables were independent risk factors for regional LNM in dMMR/MSI colorectal cancer.

**Table 3:** Risk factors for regional lymph node metastasis in dMMR colorectal cancer.

Variable	Univariate analysis		Multivariate analysis	
	OR (95% CI)	*p*-Value	OR (95% CI)	*p*-Value
Age (years)	1.03 (0.99–1.06)	0.078	1.04(1.01–1.08)	0.031
Location (left colon and rectum)	2.70 (1.16–6.28)	0.021	7.31 (2.21–24.15)	0.001
Degree of differentiation (high-grade)	4.11 (1.82–9.29)	0.001	9.53 (3.16–28.74)	<0.001
Depth of invasion (T3–T4)	6.30 (0.79–49.59)	0.081	22.20 (1.87–262.78)	0.014
Negative IHC staining of MMR proteins (except for double-negative staining of MLH1 and PMS2 or MSH2 and MSH6)	2.42 (1.01–5.80)	0.047	5.09 (1.56–16.67)	0.007

### Development and validation of a clinicopathological nomogram for predicting LNM in dMMR/MSI colorectal cancer

**Figure 1:**
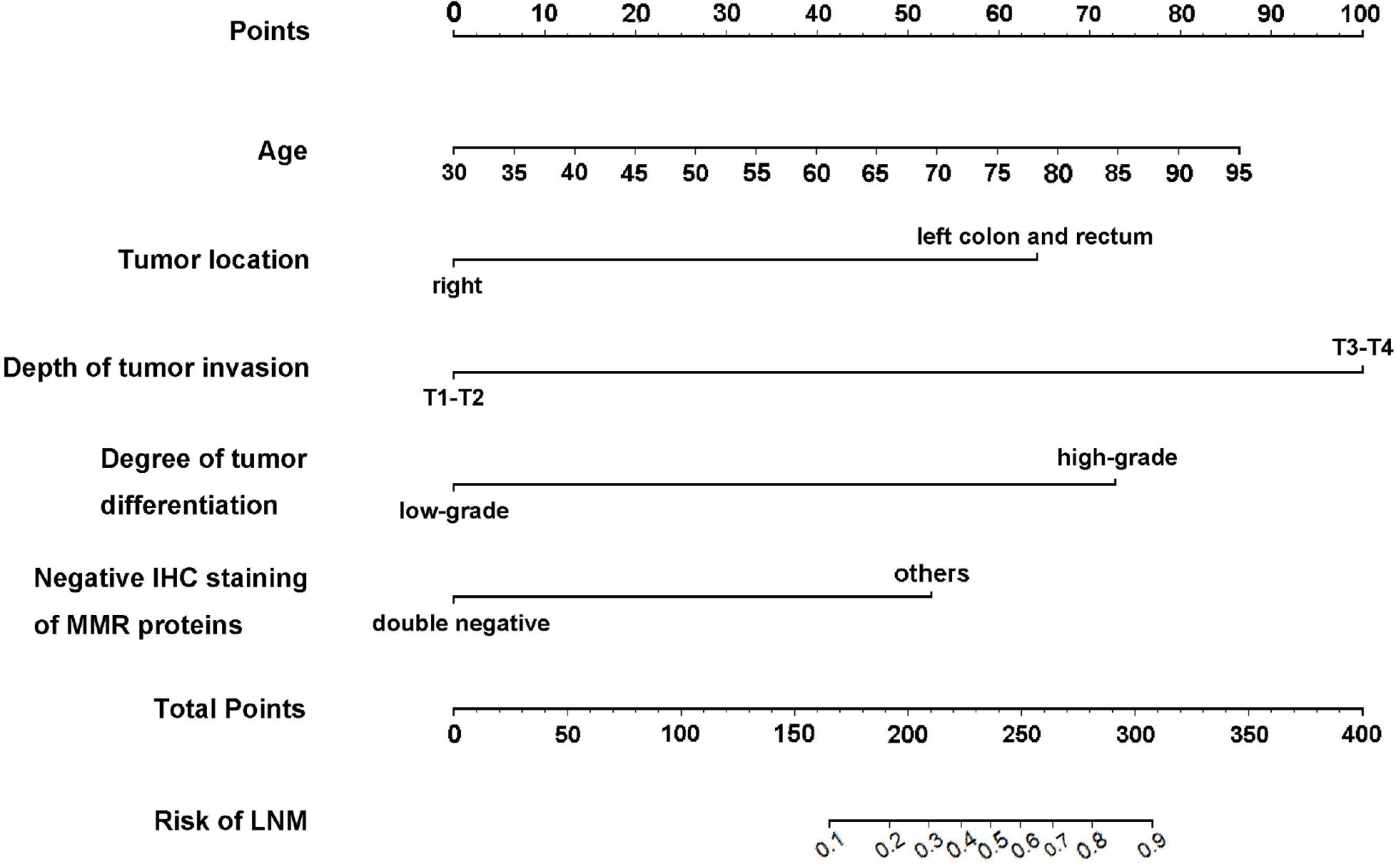
The developed clinicopathological nomogram for predicting LNM in dMMR/MSI colorectal cancer. The clinicopathological nomogram was developed by incorporating the following independent factors: age, tumor location, depth of invasion, degree of differentiation, and negative IHC staining of MMR proteins, except for the double-negative staining of MLH1 and PMS2 or MSH2 and MSH6. Clinical scores were assigned to the five independent factors and the estimated risk was calculated by summing the scores of each factor. The “Points” value represents the score of these factors, and the “Total Points” value indicates the sum of the score of all the above factors.

**Figure 2:**
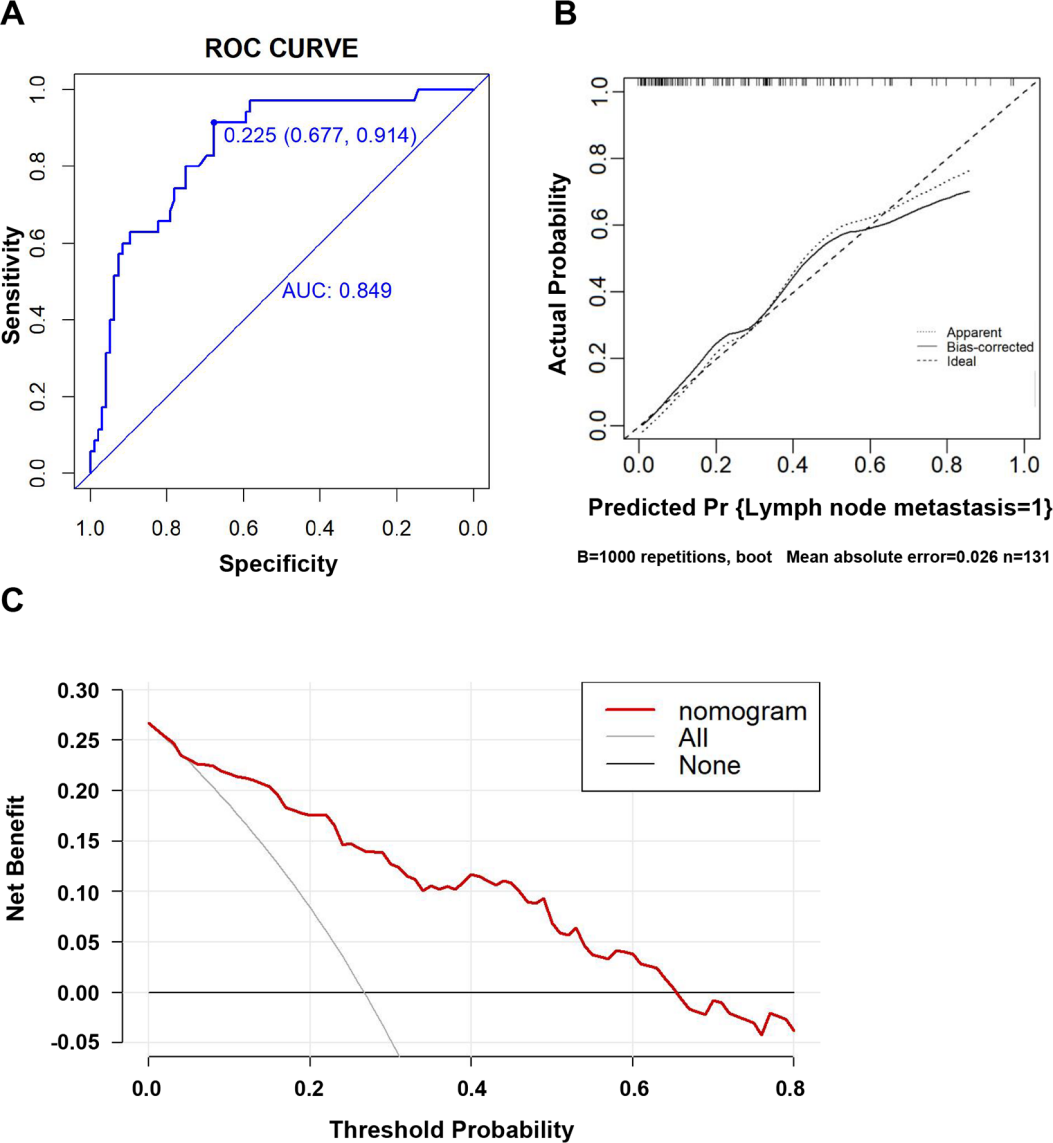
Evaluation of the clinicopathological nomogram for predicting LNM in dMMR/MSI colorectal cancer. **(A)** The receiver operating characteristic (ROC) curve was adopted to evaluate the discriminatory ability of the nomograms. The area under the ROC curve is 0.849, which is more than 0.7, suggesting reasonable discrimination. **(B)** Internal validation was performed using bootstrapping with 1,000 repetitions, shown as a calibration curve, which represents the agreement between the observed outcome and the predicted probabilities. The *y*-axis represents the actual LNM rate. The *x*-axis represents the predicted LNM risk. The diagonal line represents a perfect prediction by an ideal model. The dotted line represents the calibration performance of the nomogram on the training data. The solid line represents the performance of the nomogram after bootstrap validation (1,000 repetitions), with a corrected C-statistic and Brier score of 0.85 and 0.14, respectively. The Hosmer–Lemeshow test yielded a non-significant statistic (*p* = 0.396), which suggested that there was no departure from the perfect fit. **(C)** Results of the decision curve analysis (DCA). The *y*-axis represents the net benefit. The *x*-axis represents the threshold of a doctor’s prediction probability. The red line represents our clinicopathological nomogram. The light gray line represents the assumption that all patients have LNM. The gray line represents the assumption that no patients have LNM. If the threshold probability (Pt) of the doctor’s prediction probability is between 5% and 65%, using our nomogram to predict the regional LNM in dMMR/MSI colorectal cancer had more benefit than either the treat-all-patients scheme or the treat-none scheme. Furthermore, when Pt was greater than 65%, the benefit was lower than the effect of the treat-none scheme. The probability of LNM is higher when the predicted probability obtained from our nomogram is higher. If the threshold for predicting the probability by doctors is too high, it would result in the lymph nodes of cases that need to be cleared not being cleared, and the benefit would decrease. Therefore, the threshold of the prediction probability should be greater than 5% and not exceed 65%; that is, be between 5% and 65%.

Based on the results of the multivariate logistic regression analysis, we developed an intuitive clinicopathological nomogram for the individualized preoperative prediction of LNM in patients with dMMR/MSI CRC. The nomogram included the following five independent predictors: age, tumor location, depth of invasion, degree of differentiation, and negative IHC staining results for MMR proteins (**Figure 1**). ROC was adopted to evaluate its discrimination. The apparent performance (C-statistic) AUROC was 0.85 (95% CI: 0.78–0.92), which was greater than 0.7, indicating that the discriminatory ability was excellent (**Figure 2A**). Then, a 1,000 resampling bootstrapping calibration was conducted for internal validation. The optimism-corrected C-statistic was 0.83 (95% CI, 0.78–0.85), indicating that the nomogram retained robust discriminatory ability. The optimism-corrected C-slope was 0.70 (95% CI, 0.35–1.22), which was consistent with the expectation of the small-size sample model. CITL was near zero (0.02; 95% CI, -0.50–0.56), and the Brier score was 0.14 (95% CI, 0.09–0.17), indicating that the calibration accuracy was preserved after validation, and the predicted probability was consistent with the actual probability (**Table 4**). Moreover, the Hosmer–Lemeshow test yielded a non-significant statistic (*p* = 0.396), indicating there was no difference between the predicted values obtained by our nomogram and the observed outcomes (**Figure 2B**). Therefore, while external validation remains essential, the bootstrap-corrected metrics demonstrate reliable performance within the studied population. The result of the DCA for the nomogram is presented in **Figure 2C**. It showed that if the threshold probability of a patient was between 5% and 65%, using our clinicopathological nomogram to predict regional LNM in dMMR/MSI colorectal cancer adds more benefit than either the treat-all-patients scheme or the treat-none scheme. This indicates that the nomogram can be used for early clinical prediction of regional LNM in dMMR/MSI colorectal cancer.

**Table 4:** Apparent and internal validation performance of the prediction model, including C-statistic, calibration slope (C-slope), and calibration-in-the-large (CITL) values.

Performance measure	Apparent	Average optimism	Optimism-corrected
C-statistic	0.85	0.02	0.83 (95% CI, 0.78–0.85)
C-slope	1.00	0.30	0.70 (95% CI, 0.35–1.22)
CITL	0.00	-0.02	0.02 (95% CI, -0.50–0.56)

## Discussion

Although dMMR/MSI colorectal cancer has good RFS, the survival after relapse rate is remarkably poor. The presence of regional LNM is closely related to a high incidence of relapse and a worse prognosis. Accurately assessing the presence of regional LNM before surgery will help guide individualized treatment plans, avoid overtreatment, and reduce the occurrence of perioperative complications such as vascular and nerve damage and lymphatic leakage caused by excessive lymph node dissection, especially for patients with dMMR/MSI CRC. In this retrospective study, we used common clinicopathological indicators and investigated the risk factors for LNM in patients with dMMR/MSI CRC. Our findings suggested that a higher risk of regional LNM in dMMR/MSI colorectal cancer was associated with older age, tumor in the left colon and rectum, high-grade differentiation, T3–T4 invasion, neural invasion, vascular invasion, and negative IHC staining results for MMR proteins except for the double-negative staining of MLH1 and PMS2 or MSH2 and MSH6 (**Tables 1**-**3**). We then incorporated these factors, with the exclusion of neural invasion and vascular invasion, as these could not be detected preoperatively, and developed an intuitive clinicopathological nomogram for predicting LNM preoperatively. The nomogram had sufficient discriminability and calibration, demonstrating good clinical practicality (**Figures 1**, **2** and **Table 4**).

The degree of tumor differentiation has been considered an important factor in metastasis in patients with CRC (28, 29). In dMMR/MSI colorectal cancer, we also found that high-grade differentiation was a high-risk factor for regional LNM compared to low-grade differentiation (**Tables 2** and **3**). For pT staging, data from a Japanese study showed that the proportion of regional LNM gradually increased with an increase in pT staging. Approximately 10% of patients with stage pT1 had LNM, while more than 50% of patients with stages pT3–T4 were accompanied by LNM (20). Our data also showed that only 6.2% of the patients with stages pT1–T2 had regional LNM, while 29.6% of the patients with stages pT3–T4 had regional LNM in dMMR/MSI colorectal cancer (**Tables 2** and **3**). Therefore, high-grade differentiation and stages pT3–T4 are independent factors for regional LNM in dMMR/MSI colorectal cancer.

In the DNA MMR system, MSH2 and MSH6 together form the MutS protein complex, which can specifically recognize the errors of nucleotides during DNA replication, and subsequently activate the MutL protein complex (MLH1+PMS2) to further repair DNA strands with errors (30). dMMR function induces neoantigens and increased MSI. Due to the high cost and complex technology required for PCR-based detection of MSI, IHC detection of MMR protein is commonly used in clinical practice for screening purposes (31). In this study, we found for the first time that negative staining of MMR proteins, except for the double-negative staining of MLH1 and PMS2 or MSH2 and MSH6, was associated with a higher risk of regional LNM in dMMR/MSI colorectal cancer (**Tables 2** and **3**). The CRCs that arise via the dMMR/MSI molecular biological pathway show certain clinicopathological features, including proximal colon location, mucinous histology, infiltration by lymphocytes and a high tumor mutation burden (TMB) (8, 24, 25). Recently, Bajwa-Ten Broeke et al. (26) reported that single PMS2-deficient CRC displayed a higher tumor stage, lower CD3^+^ T-cell infiltration, and a trend towards fewer mutations in *B2M*; it may resemble pMMR CRC with regards to tumor initiation and early evolution. The loss of PMS2 appears to occur rather as a secondary event following a neoplasia-inducing variant in pMMR CRC, such as pathogenic *APC* variants. Helderman et al. also reported that *MSH6*-mutated CRC exhibited lower frequencies and mutant allele ratios across most coding microsatellites, thus leading to a relatively lower degree of MSI (27). Wang et al. (32) proposed classifying the heterogeneous MMR protein IHC pattern into the following four subgroups: “single-loss”, “MLH1/PMS2 double-loss”, “MSH2/MSH6 double-loss”, and “triple/tetra loss”, with the genetic mechanism of “single-loss” mostly associated with somatic mutations, and the others associated with germ-line mutations. Compared with the expression loss of a single MMR protein/heterodimer, a double-negative MMR protein IHC pattern is characterized by a higher level of non-synonymous variants, unstable microsatellite loci, a significant increase in TMB, and an increased number of tumor-infiltrating lymphocytes, which are also characterized by a substantial population of exhausted CD8^+^ lymphocytes (25). In our study, we found that compared with the others, the double-negative staining results for MLH1 and PMS2 or MSH2 and MSH6 were related to a lower likelihood of regional LNM, which was consistent with the above reports. Therefore, we infer that, except for the double-negative IHC pattern, other negative staining results for MMR proteins are more likely to be associated with a higher probability of regional LNM in dMMR/MSI colorectal cancer.

One advantage of our nomogram is that it is constructed based on the clinical pathological variables widely used in clinical CRC staging. Therefore, it can be used in resource-limited settings where clinicians and pathologists may still have all the data required to effectively use it preoperatively. Due to the low occurrence of dMMR/MSI tumors, the sample size in our study was limited, as in the previous reports (33, 34). We implemented a rigorous 1,000-replicate bootstrap internal validation, which demonstrated stable performance metrics despite sample size constraints [the optimism-corrected C-statistic was 0.83 vs. apparent value of 0.85; CITL of 0.02 vs. apparent value of 0; the Brier score was 0.14 (95% CI, 0.09-0.17)], confirming the adequate accuracy and generalizability of the model within the studied population. These results align with recommendations for internal validation using bootstrap resampling to mitigate overfitting risks in prediction models (22). The decision curve showed that if the threshold probability was between 5% and 65%, using our clinicopathological nomogram to predict regional LNM in dMMR/MSI CRC adds more benefit than either the treat-all-patients scheme or the treat-none scheme. Therefore, in view of the preoperative prediction of regional LNM in patients with dMMR/MSI CRC, our nomogram is an important additional source of information to guide early interventions, such as lymphadenectomy, and has good value for practical clinical decision-making.

This study has limitations. First, due to the low prevalence of dMMR/MSI tumors, the sample size in this retrospective study was relatively small. Although the bootstrap-corrected metrics provide estimates of model reliability, external validation studies should be performed in subsequent research, preferably by independent investigators, to evaluate model performance (22). Second, in order to accurately identify risk factors and make predictions, factors such as T staging in the analysis still come from postoperative pathological results. The combination of preoperative CT or MRI imaging examination, endoscopy, and histological examination can relatively accurately determine the patient’s T stage and tissue differentiation. Our next step will be to attempt to construct a multidimensional evaluation system that integrates a patient’s clinical pathological information, imaging information, and tumor biology information, further improving the accuracy of the preoperative prediction of regional LNM in dMMR/MSI colorectal cancer so as to provide a reference for clinical treatment decision-making.

In summary, for patients with dMMR/MSI CRC, age, tumor location, depth of invasion, degree of differentiation, and a negative IHC pattern for MMR proteins, except for the double-negative staining of MLH1 and PMS2 or MSH2 and MSH6, are associated with regional LNM independently. Our nomogram can be used to effectively predict regional LNM and develop treatment plans, such as lymphadenectomy, for different subgroups of patients with dMMR/MSI CRC.

## Data Availability

The original contributions presented in the study are included in the article/Supplementary Material. Further inquiries can be directed to the corresponding authors.
